# Discontinuities in soil strength contribute to destabilization of nutrient-enriched creeks

**DOI:** 10.1002/ecs2.2329

**Published:** 2018-08

**Authors:** Cathleen Wigand, Elizabeth B. Watson, Rose Martin, David S. Johnson, R. Scott Warren, Alana Hanson, Earl Davey, Roxanne Johnson, Linda Deegan

**Affiliations:** 1 Atlantic Ecology Division, ORD-NHEERL, U. S. Environmental Protection Agency, 27 Tarzwell Drive, Narragansett, Rhode Island 02882 USA; 2 Patrick Center for Environmental Research, The Academy of Natural Sciences, Drexel University, 1900 Benjamin Franklin Parkway, Philadelphia, Pennsylvania 19103 USA; 3 Virginia Institute of Marine Science, College of William & Mary, 1375 Greate Road, Gloucester Point, Virginia 23062 USA; 4 Department of Botany, Connecticut College, 270 Mohegan Avenue, New London, Connecticut 06320 USA; 5 Marine Biological Laboratory, Woods Hole Research Center, 149 Woods Hole Road, Falmouth, Massachusetts 02540 USA

**Keywords:** eutrophication, marsh loss, sea level rise, soil shear strength, wetland soil

## Abstract

In a whole-ecosystem, nutrient addition experiment in the Plum Island Sound Estuary (Massachusetts), we tested the effects of nitrogen enrichment on the carbon and nitrogen contents, respiration, and strength of marsh soils. We measured soil shear strength within and across vegetation zones. We found significantly higher soil percent organic matter, carbon, and nitrogen in the long-term enriched marshes and higher soil respiration rates with longer duration of enrichment. The soil strength was similar in magnitude across depths and vegetation zones in the reference creeks, but showed signs of significant nutrient-mediated alteration in enriched creeks where shear strength at rooting depths of the low marsh–high marsh interface zone was significantly lower than at the sub-rooting depths or in the creek bank vegetation zone. To more closely examine the soil strength of the rooting (10–30 cm) and sub-rooting (40–60 cm) depths in the interface and creek bank vegetation zones, we calculated a vertical shear strength differential between these depths. We found significantly lower differentials in shear strength (rooting depth < sub-rooting depths) in the enriched creeks and in the interface zones. The discontinuities in the vertical and horizontal shear strength across the enriched marshes may contribute to observed fracturing and slumping occurring in the marsh systems. Tide gauge data also showed a pattern of rapid sea level rise for the period of the study, and changes in plant distribution patterns were indicative of increased flooding. Longer exposure times to nutrient-enriched waters and increased hydraulic energy associated with sea level rise may exacerbate creek bank sloughing. Additional research is needed, however, to better understand the interactions of nutrient enrichment and sea level rise on soil shear strength and stability of tidal salt marshes.

## Introduction

Population growth and human activities, especially along the coast, result in increasing loads of reactive nitrogen from land to coastal waters (e.g., Deegan 2002, Galloway et al. 2004, Howarth and Marino 2006). Although many marsh studies have demonstrated enhanced plant growth under high-nutrient inputs (e.g., Valiela et al. 1975, Anisfeld and Hill 2012, Fox et al. 2012, Morris et al. 2013a, b, Wigand et al. 2015, Davis et al. 2017), human wastewater, sewage effluent, and agricultural runoff are sometimes implicated as causes for coastal marsh loss (Turner et al. 2009, Turner 2011, Deegan et al. 2012, Wigand et al.(2014) One factor that strongly mediates impacts of nutrient additions on coastal wetland is soil composition. Belowground salt marsh loss due to nutrient additions has been reported for some systems dominated by in situ organic matter (OM) accumulation (e.g., Valiela et al. 1976, Turner et al. 2009, Turner 2011, Deegan et al. 2012, Wigand et al. 2014). In contrast, in marsh systems dominated by sediment inputs, above- and belowground productivity and peat buildup can be enhanced by nutrient subsidies (e.g., Morris et al. 2013b, Wigand et al. 2015, Davis et al. 2017).

Exacerbating coastal eutrophication are high rates of relative sea level rise, which are reported to be 3–4 times greater in the Northeast United States than the global average (Church and White 2006, Boon 2012, Sallenger et al. 2012, Calafat and Chambers 2013). In recent years, sea level rise is reportedly accelerating and considered a key factor driving large-scale marsh loss in some parts of New England (Watson et al. 2014, 2017, Weston 2014, Raposa et al. 2017). Since both nutrient enrichment and rapid sea level rise can cause marsh loss (Kirwan and Megonigal 2013, Watson et al. 2014, Wong et al. (2015) in the present study we consider the possible effects of increased inundation associated with accelerated sea level rise to the creek systems in addition to nutrient treatments in a whole-ecosystem enrichment experiment.

Nutrient enrichment caused the destabilization of salt marshes in a long-term, whole-ecosystem experiment in the Plum Island Sound Estuary (Massachusetts), located in the Northeast United States (Deegan et al. 2012). The marsh response to long-term nitrogen enrichment included significant landscape fracturing (structural failure resulting in long cracks along creek banks) and the subsequent slumping of the marsh into the creek, which was attributed, in part, to a significant reduction in the belowground live biomass and the drag by tidal currents (Deegan et al. 2012). Belowground roots and rhizomes in marshes can increase the strength of soils by providing mechanical reinforcement, which is related to the diameter and density of belowground structures and rooting depth (Howes et al. 2010). Nutrient enrichment is proposed to reduce belowground biomass, increase fine OM, and increase soil decomposition rates. More decomposed or sapric marsh soils can reduce soil shear strength, causing marshes to be more susceptible to erosional processes (Swarzenski et al. 2008, Turner et al. 2009, Turner2011).

In this study, which was part of the Plum Island Sound Estuary enrichment experiment (Deegan et al. 2012), we examined soil shear strength, respiration, and nutrient content in the enriched and reference creeks to better understand possible causes for marsh fracturing and slumping. We hypothesized that while nutrient enrichment could cause increased sapric OM, nitrogen content, and respiration rates in marsh soils, it might also cause decreased soil shear strength across the marsh landscape, possibly due to the previously reported significant reduction in belowground biomass and associated fibric soils (Deegan et al. 2012) and elevated rates of belowground OM mineralization associated with nitrogen processing (Koop-Jackobsen and Giblin 2010). Because sea level rise is known to alter the extent and frequency of flooding of coastal marshes and alter plant community structure in the Northeast United States (e.g., Warren and Niering 1993, Roman et al. 1997, Donnelly and Bertness 2001, Raposa et al. 2017), we calculated yearly tidal flooding patterns for the study period and plant species cover to examine whether there were shifts in dominant species toward those favored by wetter soils. We evaluate the results of our study within the context of nutrient enrichment and sea level rise, as well as the earlier reported significant increase in creek bank fracturing and slumping at the enriched sites (Deegan et al. 2012).

## Methods

### Site description and experimental design

The study was conducted at six first-order tidal creeks located within the Plum Island Estuary (42°44′ N 70°52′ W), previously described in detail (Deegan et al. 2007, 2012, Johnson et al. (2016) Two creeks were fertilized for nine years (beginning in 2004), one creek for four years (beginning in 2009), and the remaining three creeks were reference creeks. Hereafter, we will refer to the nine-year enriched creeks (*n* = 2) as the long-term enriched creeks and the four-year enriched creek (*n* = 1) as the short-term enriched creek. Two reference creeks were paired with the long-term enriched creeks and studied for 9 yr, while one reference creek was paired with the short-term enriched creek and studied for 4 yr. The ecosystem scale of the experiment necessitated low replication at the creek level.

The creek systems had similar length (300–500 m), volume (4.1–7.5 × 10^6^ L), landscape position, and physicochemistry (Deegan et al. 2007, 2012, Johnson et al. 2016). Marsh vegetation was characteristic of a typical New England salt marsh with a high marsh (i.e., elevations above mean high water [MHW]) dominated by *Spartina patens, Distichlis spicata*, and stunted *Spartina alterniflora* and a low marsh dominated by tall *S. alterniflora*, primarily growing along the creek banks (Johnson et al. 2016). Enriched creeks received nutrients twice daily via flooding tides during the growing season (approximately 120 d; 15 May–15 September), over an area of about 30,000 m^2^ of marsh per experimental creek (Deegan et al. 2012). The target concentration for the water column flooding the enriched marshes was 70–100 μmol/L NO_3_ (as NaNO_3_), which was 10–15 times greater than Plum Island Sound background levels (Johnson et al. 2016). Initially, PO_4_ (as NaH_2_PO_4_) was added to target 5–7 μmol/L, approximately achieving a 15:1 Red-field ratio to avoid secondary phosphorus (P) limitation; however, P addition was discontinued after 2010, as earlier dissolved nutrient analyses indicated that P was naturally in excess (Johnson et al. 2016).

### Soil shear strength

To test whether nutrient enrichment reduces soil strength, we examined the soil shear strength of the marsh landscape vertically with depth and horizontally across vegetation zones (low marsh; interface between the high and low marsh; high marsh) of the reference and enriched creeks. The shear strength of the marsh soils reflects the resistance to shearing stresses afforded by the cohesion and frictional resistance of the soil constituents and is presumed to also be an indicator of the integrity of the root and soil matrix in coastal marshes (Turner et al. 2009, 2017, Howes et al. 2010). Shear strength has also been used as an indicator of marsh soil decomposition, the less torque that is needed to shear the soil, the more decomposed that soil is considered (Swarzenski et al. 2008). A field-vane shear tester (AMS part 59020, American Falls, Idaho) similar to the instrument and methods reported in other marsh studies (Swarzenski et al. 2008, Turner et al. 2009, Howes et al. 2010, Turner 2011, Graham and Mendelssohn 2014) was used to measure the minimum shear strength in kilopascals (kPa) required to force soil failure at nine depths, beginning at a depth of 10 cm, and in increments of 10 cm thereafter. The shear strength values obtained with a field-vane may overestimate marsh soil strength because of strain rate, anisotropy within the soil, and rod friction, but field-vane measures of shear strength in marsh systems are useful and acceptable for comparative purposes (e.g., Turner et al. 2009, Howes et al. 2010, Turner 2011, Graham and Mendelssohn 2014). For statistical analyses, three depth increments (averaged across 10 cm intervals) were examined: 10–30, 40–60, and 70–90 cm. Shear strength profiles were only measured in intact areas of the marsh, not in fractured areas or on clumps of marsh in the creeks.

Vertical profiles of shear strength were carried out ± 2 h of low tide to allow for comparisons of marsh soil strength at similar tide heights among sites in August 2012. The soil strength measures were carried out 9 yr after the initiation of nutrient additions at the long-term enriched and 4 yr after initiation of nutrient additions at the shortterm enriched sites. Transects at each site were sampled once. A point along the creek bank was haphazardly located *ca*. midway between the mouth and terminus of each creek. The soil shear strength transects were oriented perpendicular to the creek at this point. Additional transects, ~15 m apart, also set perpendicular to the creek were established on both sides of the first. A total of four transects were set along each long-term enriched (*n* = 2) and reference (*n* = 2) creeks with eight transects in the short-term enriched (*n* = 1) and paired reference (*n* = 1) creek.

Three zones on the marsh landscape were sampled along each transect, first in the low marsh (i.e., creek bank tall *S. alterniflora*), then at the interface zone between the tall *S. alterniflora* and the high marsh, and, third, in the high marsh (i.e., dominated by *S. patens*). The tall *S. alterniflora* occurred in an approximate 2–3 m wide zone along the creek bank, and the interface zone was a narrow (about 1 m in width) band composed of *S. alterniflora, S. patens, D. spicata*, and occasionally small patches of the annual *Atriplex patula.* Vertical profiles were sampled in the tall *S. alterniflora* about 1 m back from the creek bank edge, in the approximate center of the interface zone, and one meter landward from the interface zone in the high marsh. Collectively these providing a total of twelve vertical profiles of soil strength per long-term enriched and reference creeks and 24 vertical profiles per shortterm enriched and reference creek.

To more closely examine the soil shear strength associated with the active rooting (10–30 cm) and sub-rooting (40–60 cm) depths of marsh zones with *S. alterniflora* (Valiela et al. 1976, Howes et al. 2010, Graham and Mendelssohn 2014), we calculated a vertical shear strength differential between these depth intervals for the creek bank and interface zones. For each location sampled along the transects in these two zones, the 10–30 cm depth soil shear strength was subtracted from the 40 to 60 cm soil shear strength to calculate a vertical differential. We presumed that the vertical shear strength differential was associated with the stability of the marsh landscape and that marshes with positive or nearpositive shear strength differentials (rooting depth soil strength > sub-rooting depth soil strength) would be more resistant to the fracturing phenomenon, first reported in Deegan et al. (2012). For the horizontal plane, we hypothesized that low horizontal discontinuities in soil shear strength would be more resistant to fracturing, as downslope forces are greater along the interface and creek bank zones.

### Soil respiration

To test whether nutrient enrichment causes elevated soil respiration, we measured soil carbon dioxide (CO_2_) emissions in 2011 at the long-term enriched and reference marshes and in 2012 at the short-term enriched and reference marshes during summer (July−August), when maximum soil respiration rates were expected (Wigand et al. 2009). At all sites, we measured soil respiration rates with a Li-Cor (8100) CO_2_ flux system and dome using standard methods (e.g., Howes et al. 1985, Wigand et al. 2009). We placed PVC collars (10 cm diameter) in bare areas between tall *S. alterniflora* culms, near low tide, at least 15 min before in situ sampling was conducted. The instrument uses an infrared detector to measure changes in CO_2_ in the dome during 5-min incubations.

At the long-term enriched and reference sites, we measured CO_2_ emissions in the low marsh at creek bank locations along two transects in each creek earlier established for the soil shear strength measures. Five replicates of soil respiration were sampled about 1 m apart at each location to account for possible high spatial heterogeneity and averaged for statistical analyses.

We sampled CO_2_ emissions at eight creek bank locations in each short-term enriched and reference creek to achieve greater coverage of the low marsh. Creek bank locations were along the same eight transects established for the soil shear strength measures.

### Soil nutrient content

We analyzed soil OM, percent carbon, and nitrogen on cores previously collected in 2010 from the long-term enriched and reference creeks (Deegan et al. 2012,: *n* = 10 cores per creek or 20 cores per treatment). We sampled soil plugs (not sieved) at four depths: 0–5, 5–10, 10–20, and 20–30 cm in each core; ground them with a mortar and pestle; and analyzed for % C and % N contents on a Carlo Erba NA 1500 NCS elemental analyzer. We dried a separate soil sample from each depth at 105°C; these were then ashed at 550°C for 4 h to determine the percent soil OM using loss-on-ignition methods (Heiri et al. 1999). We did not measure soil nutrient content in the short-term enriched creeks.

### Sea level rise and plant cover

Using NOAA tide gauge data, we calculated yearly tidal flooding patterns for 2002–2011, a time-period spanning before and during the present study. Monthly water level data were downloaded from the NOAA COOPS Web site (http://tidesandcurrents.noaa.gov) for tide gauges south (Boston, Massachusetts) and north (Portland, Maine) of the Plum Island Estuary. Summer (May–September) MHW (m above station datum) and mean sea level (MSL, m above station datum) at Boston and Portland were plotted for 2002–2011.

Another indicator of sea level rise and increasing inundation is the composition of the high marsh plant community, with more frequently flooded marsh soils associated with a decline in dominance of *S. patens* with a concomitant increase in *D. spicata* cover, and in the low marsh, an increase in cover of *S. alterniflora* (Warren and Niering 1993, Roman et al. 1997, Raposa et al. 2017). Therefore, relationships of plant species cover in the high and low marsh of the combined long-term reference and enriched creeks over the time period of the study were examined for the aforementioned vegetation patterns of change associated with increasing inundation.

To assess the vegetation in the long-term enriched and reference marshes, visual cover estimates, previously reported (Johnson et al. 2016), were made for all plant species in July (2004–2009 and 2011) within contiguous 1-m^2^ plots along six transects, three each for all enriched and reference creeks. Transects were set normal to creek banks and extended 1–4 m down to the lower limit of *S. alterniflora* and 45–50 m back, onto the high marsh Deegan et al. 2007, Johnson et al. 2016). Percent cover was determined for *S. patens* and *D. spicata* (mean of all plots of the high marsh of each transect) and tall *S. alterniflora* (mean of all low and high marsh plots along each transect) for each year (2004–2009 and 2011). We do not report the vegetation patterns in the short-term enriched and reference creeks.

### Data analysis

We used linear mixed effects models to analyze the effects of nutrient enrichment, vegetation zone, and depth (when indicated) on soil shear strength; vertical shear strength differentials; and soil C, N, and C:N ratios. We specified enrichment treatment, vegetation zone, and depth as fixed effects, and site pairs (short- and long-term enrichment treatments and their respective references) as random effects in the models to account for non-independence of sampling (e.g., shear strength measurements at different depths at the same location; different vegetation zone locations along the same transect) where necessary. To test for effects of nutrient enrichment on soil respiration while accounting for measurements performed during different years, we specified nutrient levels (enriched, reference) as a fixed effect and blocked sites by long-term or short-term pair (specified as a random effect). For the long-term enriched and reference creeks, we averaged replicates (*n* = 5) of soil respiration at sampling locations (*n* = 2 per creek or 4 per treatment) for statistical analyses. We performed *t*-tests before pooling long-term enrichment or reference treatments to ensure no differences were present by chance. To confirm that assumptions of homoscedasticity and normality were met, we examined residual plots. To obtain *P*-values to assess significance of the effect of enrichment treatment, vegetation zone, and depth on soil metrics, we performed likelihood ratio tests of full models against models with the fixed effect of interest removed. To test for interactive effects of treatment, vegetation zone, and depth on soil metrics, we compared models with and without an interaction term using likelihood ratio tests. We used the *lme4* package for the linear mixed effects analyses and regression analyses of plant species cover data to examine for significant patterns of change over years (R Development Core Team 2012). We performed all statistical analyses in R and interpreted significant differences at α = 0.05 (R Development Core Team 2012).

## Results

### Soil shear strength and respiration

**Fig. 1.**
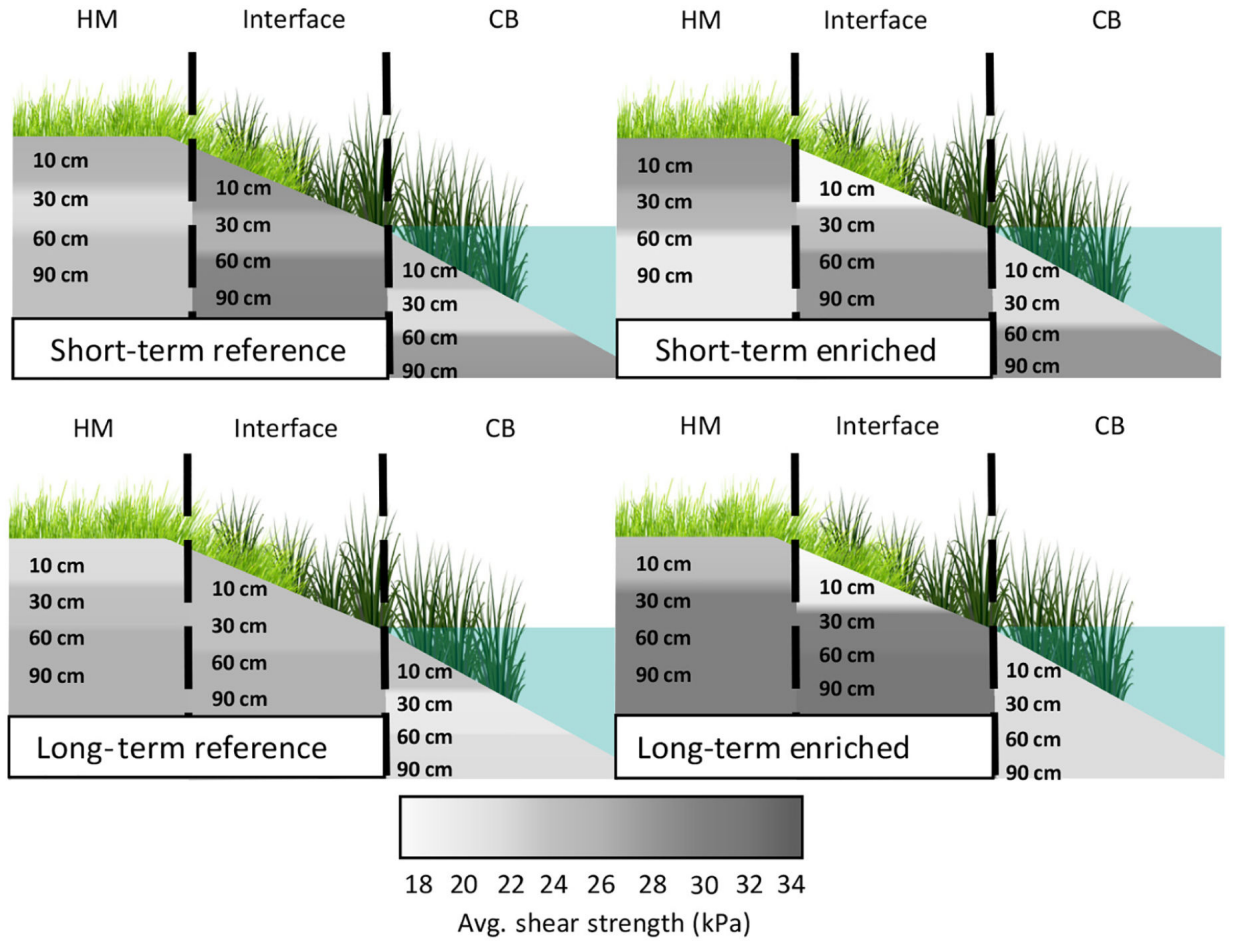
Visual representation of average shear strengths for high marsh (HM), interface, and creek bank (CB) vegetation zones at depth intervals of 10–30, 40–60, and 70–90 cm for the short- and long-term enrichment and reference creeks.

Soil shear strength of the long-term enriched creeks at the 10–30 cm depth in the interface zone was significantly lower than the soil strength at the 40–60 and 70–90 cm depth intervals of the interface and high marsh zones ($\chi_{2}^{2}8=48.0$, *P* = 0.01; Table 1; Fig. 1). The highest soil shear strength values were measured at depth intervals of 40–60 cm and 70–90 cm in the interface and high marsh zones in the long-term enriched creeks (Table 1; Fig. 1). In contrast, the soil strength was similar in magnitude across depths and vegetation zones at the reference creeks (Table 1).

In both the enriched and reference creeks, there was a trend of lower soil shear strength in the low marsh zone compared with the interface and high marsh zones ($\chi_{2}^{2}=5.53$, *P* = 0.06; Table 1; Fig. 1), significant at the 40–60 cm depth (vegetation zone × depth interaction; low marsh < interface ≤ high marsh; $\chi_{4}^{2}=11.28$, *P* = 0.02; Table 1). A main effect of depth revealed that the 10–30 cm depth was significantly lower than the 70–90 cm depth ($\chi_{2}^{2}=8.55$, *P* = 0.01).

**Fig. 2.**
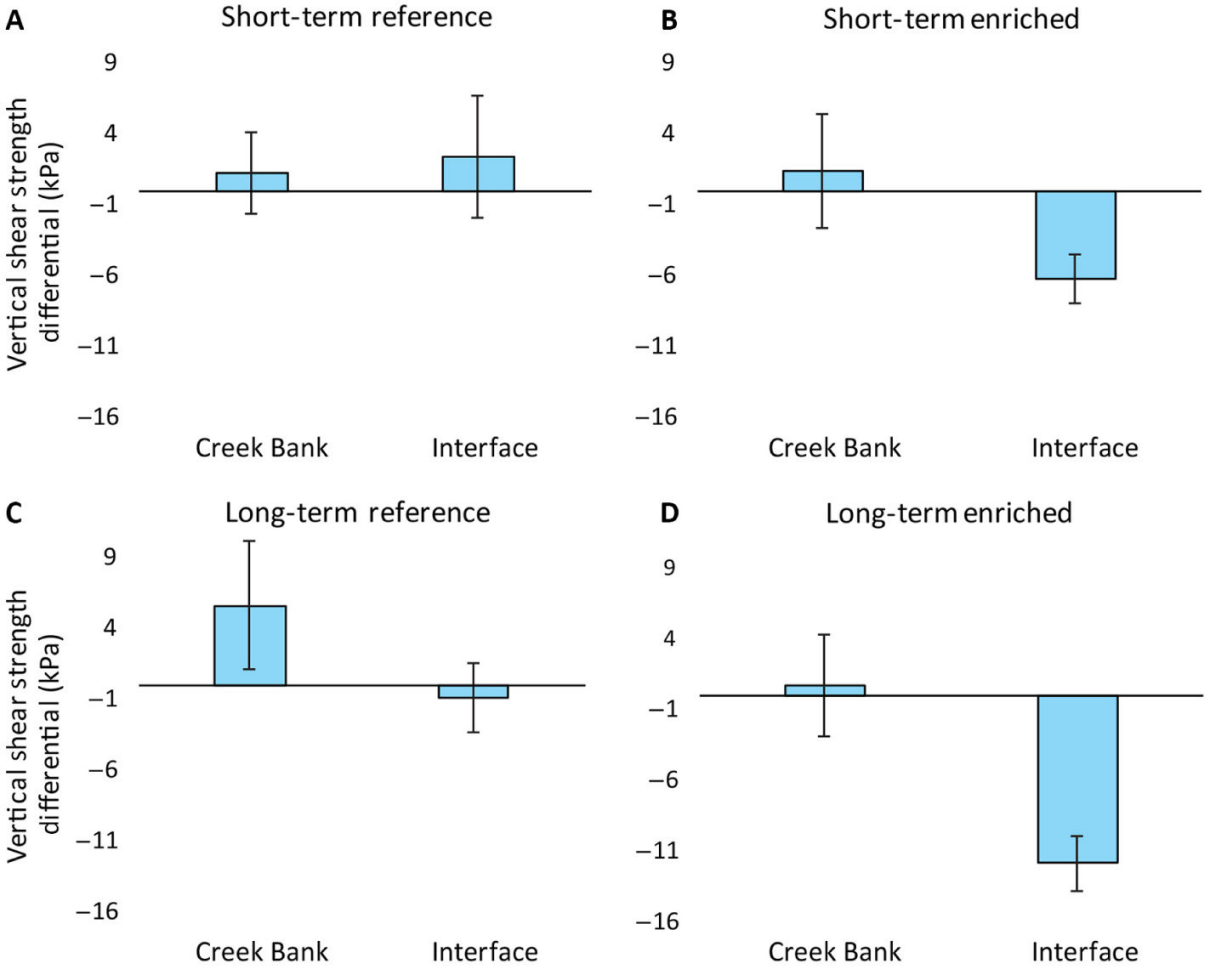
Vertical shear strength differentials for short- and long-term reference (A, C) and enriched (B, D) sites at creek bank and interface (between tall *Spartina alterniflora* and high marsh) vegetation zones. Positive means indicate stronger root zone (10–30 cm depth) shear strengths than sub-root zone (40–60 cm depth) soil, while negative means indicate the opposite pattern.

When enrichment duration (short- and longterm) was included in models, the shear strength vertical differential of the interface vegetation (−4.1 ± 2.7 kPa; rooting zone shear strength < sub-root shear strength) was significantly lower than that of the creek bank (2.3 ± 0.98 kPa, $\chi_{1}^{2}=7.25$, *P* = 0.007). In addition, there was a trend of lower vertical shear strength differentials with enrichment ($\chi_{3}^{2}=7.26$, *P* = 0.06, Fig. 2). There was no significant interaction between nutrient enrichment and marsh vegetation zone ($\chi_{3}^{2}=4.81$, *P* = 0.19). However, if the short- and long-term enrichment treatments are pooled (to form two treatment groups: enriched and non-enriched), there are significant enrichment ($\chi_{1}^{2}=6.3$, *P* = 0.01) and vegetation zone ($\chi_{1}^{2}=7.1$, *P* = 0.007; interface < creek bank) effects, although there was no statistically significant interaction between nutrient enrichment and vegetation ($\chi_{1}^{2}=2.6$, *P* = 0.10). The vertical shear strength differential of the combined enrichment treatments was −3.93 ± 1.70 kPa and significantly lower than the combined reference differential, which was 2.17 ± 1.76 kPa.

**Fig. 3.**
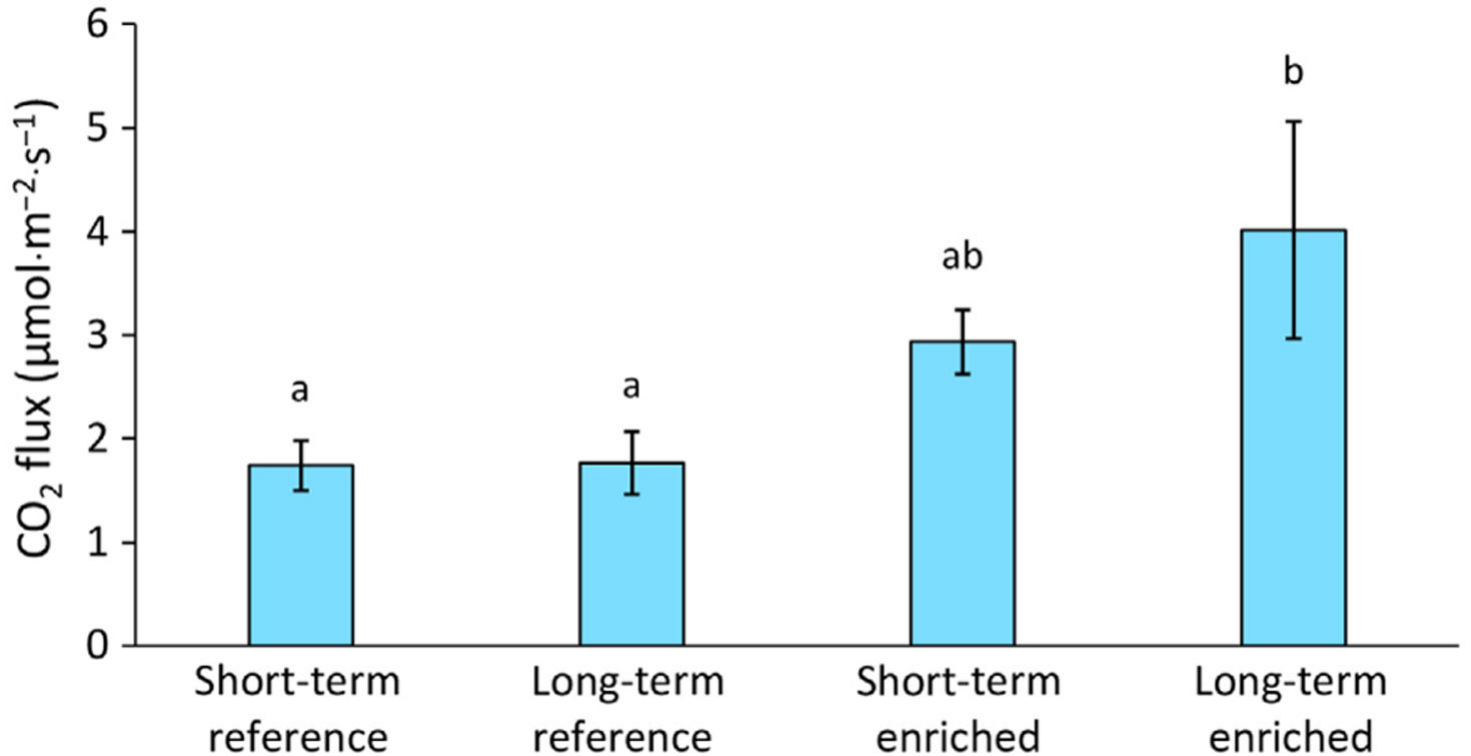
Soil respiration rates for short- and long-term reference and nutrient-enriched sites. Standard error bars are shown. Letters represent results of Tukey HSD tests. Bars sharing the same letter are not significantly different.

Nutrient enrichment had significant effects on soil respiration. The long-term enriched creeks had 27% greater soil respiration (4.02 ± 1.05 μmol·m^−2^·s^−1^) than the short-term enriched creek (2.94 ± 1.05 μmol·m^−2^·s^−1^) and over 55% greater soil respiration than the short-term reference creek (1.74 ± 0.24 μmol·m^−2^·s^−1^) and long-term reference creeks (1.77 ± 0.30 μmol·m^−2^·s^−1^) ($\chi_{3}^{2}=16.03$, *P* = 0.001; Fig. 3). The trend toward intermediate values at the short-term enriched creek suggests that these shifts reveal soil changes over time in response to nutrient enrichment.

### Soil nutrient content

**Fig. 4.**
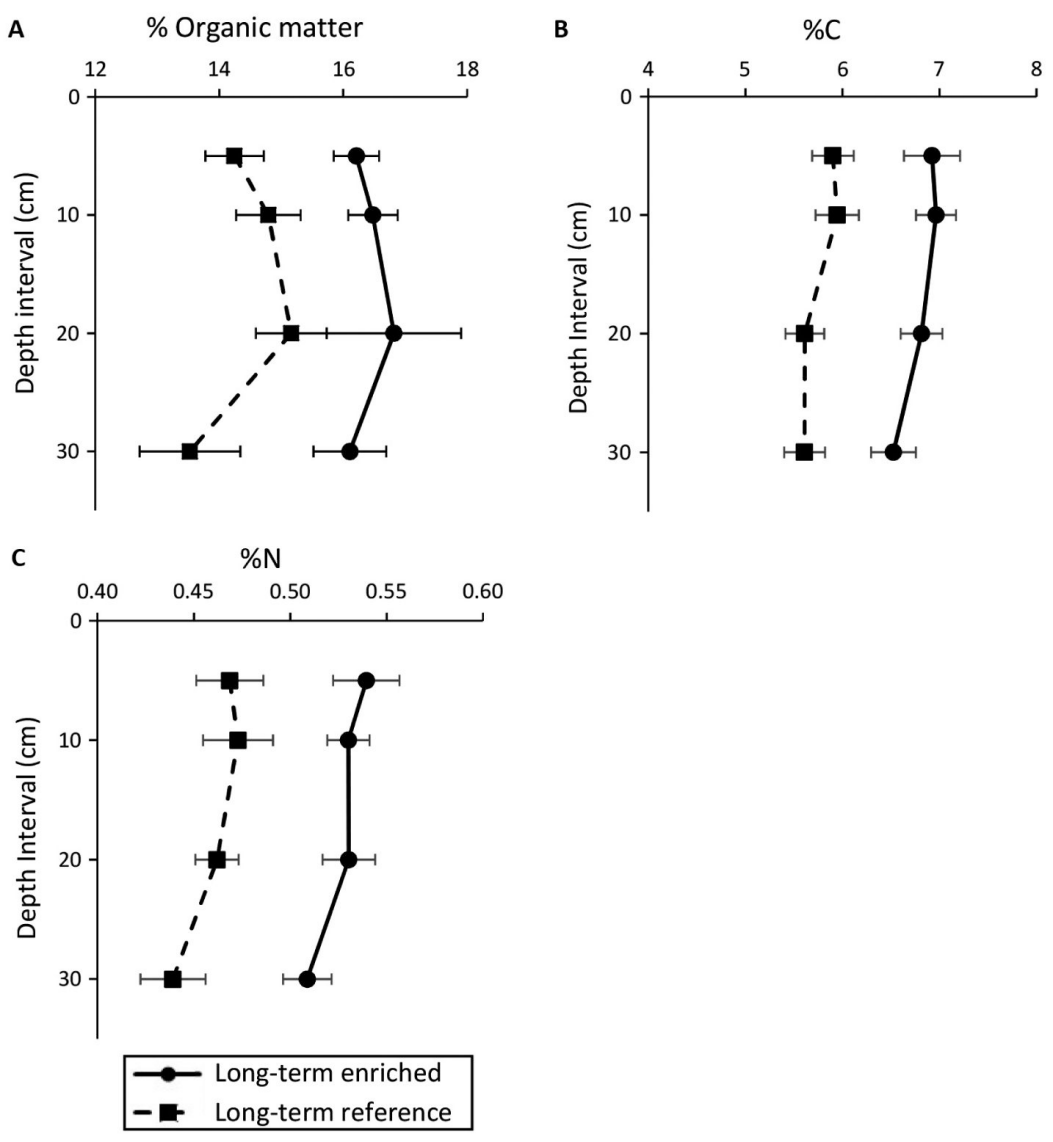
Soil percent organic matter (A), percent carbon (B), and percent nitrogen (C) from the long-term reference (dashed line) and enriched (solid line) sites. Standard error bars are shown.

The long-term enriched marsh had significantly greater mean soil OM (16.4% ± 0.59) than the long-term reference marsh (14.4% ± 0.61; $\chi_{1}^{2}=5.49$, *P* = 0.02; Fig. 4A). There was no main effect of depth or site by depth interaction on soil OM. Similarly, the long-term enrichment marsh had significantly higher soil % C (6.9% ± 0.24 enriched vs. 6.0% ± 0.21 reference; $\chi_{1}^{2}=19.52$, *P* < 0.001) and soil % N (0.53% ± 0.01 enriched vs. 0.47% ± 0.02 reference; $\chi_{1}^{2}=13.1$, *P* = 0.0002; Fig. 4B, C) than the long-term reference marsh, maintaining similar C:N molar ratios between treatments (15.08 ± 0.24 enriched and 14.89 ± 0.33 reference). There was no main effect of depth or site by depth interaction on soil % N or % C.

### Sea level rise and plant cover

**Fig. 5.**
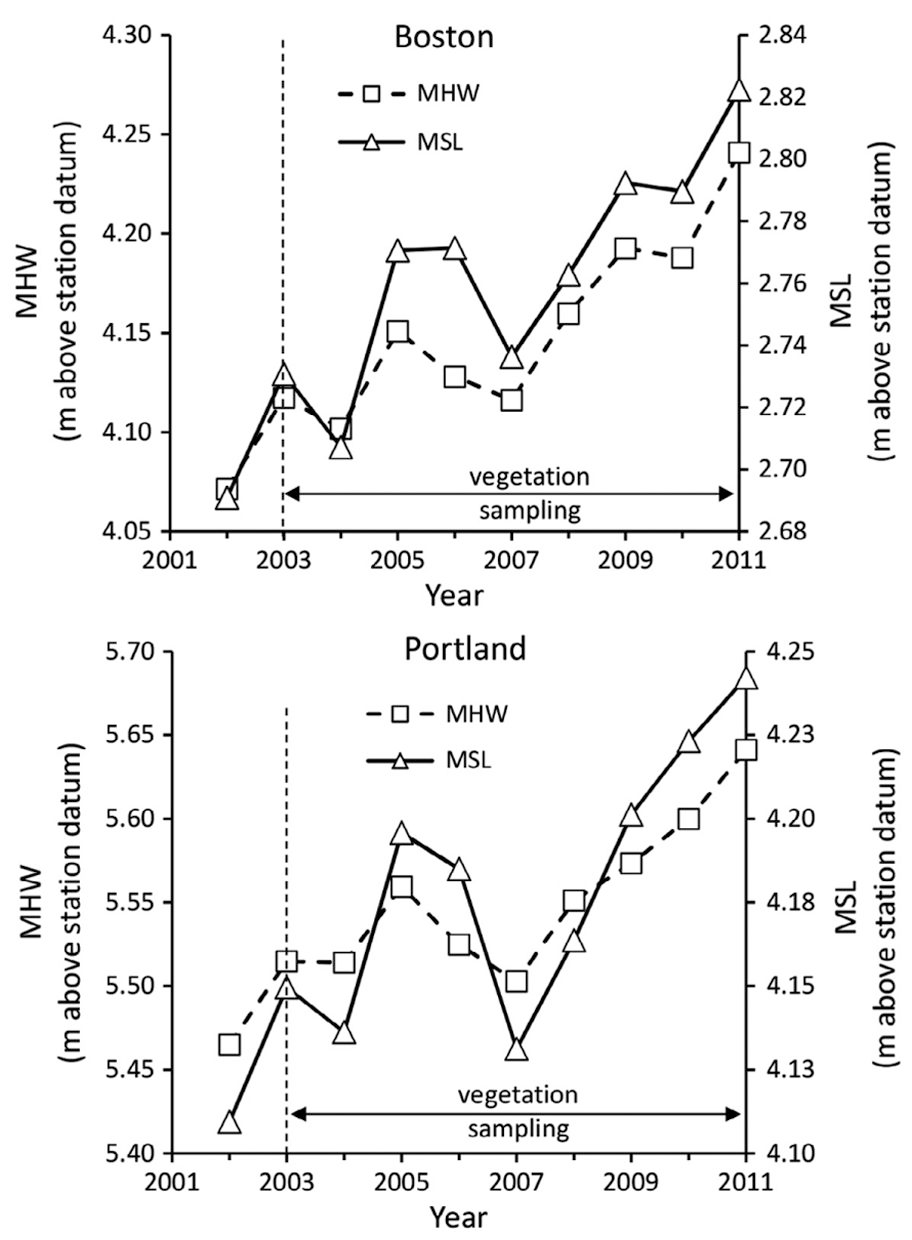
Summer (May–September) mean high water (MHW, m above station datum) and mean sea level (MSL, m above station datum) at Boston and Portland. Period of vegetation sampling for the TIDE nutrient enrichment experiment (2003–2011; Deegan et al. 2012) is indicated by horizontal arrow.

The sea level data for this coastal area suggested a pattern of rapid sea level rise for the period of the present study (Fig. 5). From 2003 to 2011 the Boston, Massachusetts (~40 km to the south) summer yearly MHW increased 12.3 cm and the mean yearly sea level (MSL) by 9.2 cm, and for Portland, Maine (*ca.* 115 km to the north) MHW increased by 12.6 cm and MSL by 9.3 cm.

**Fig. 6.**
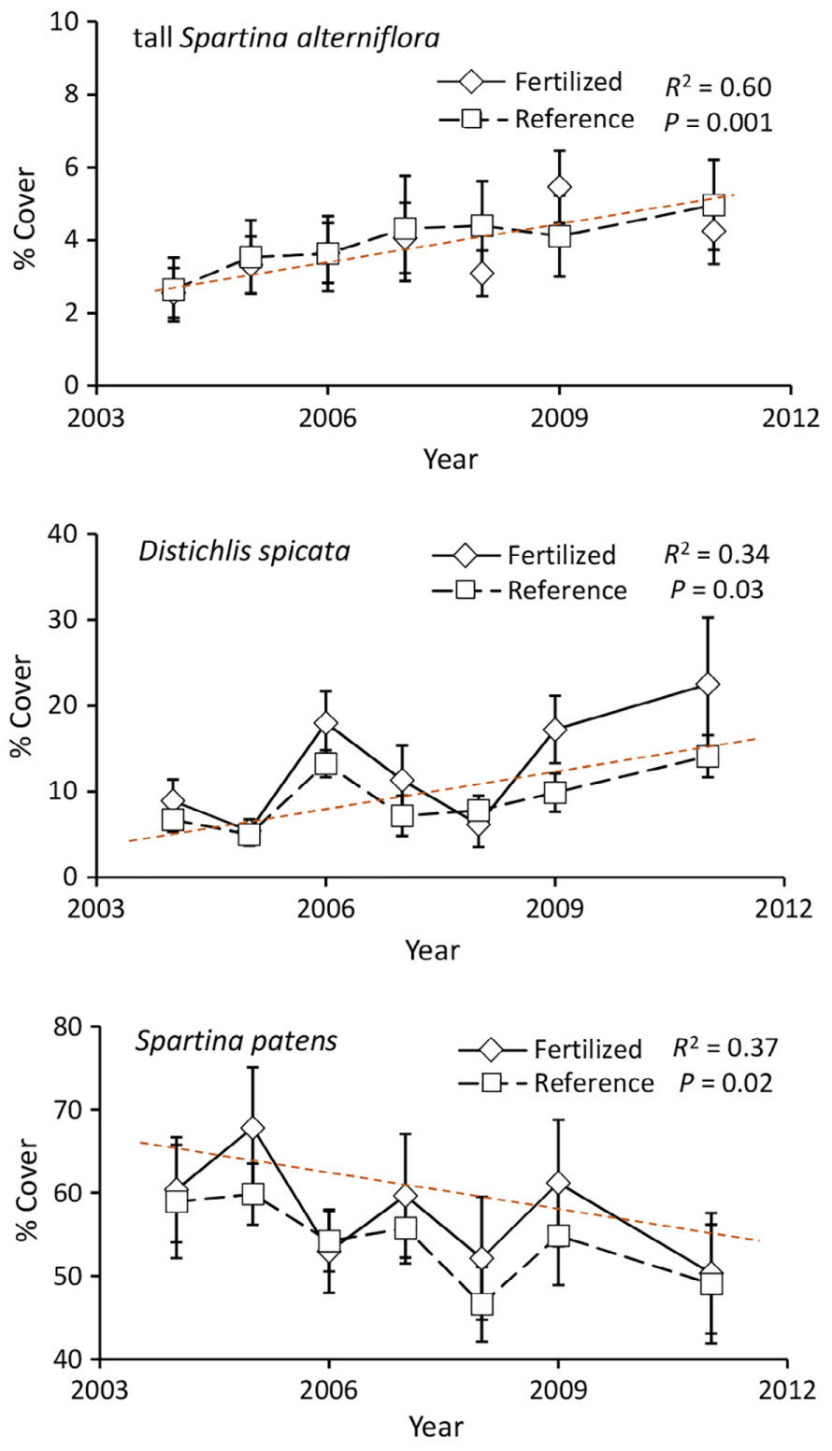
Cover changes in the dominant high marsh grasses, *Spartina patens*, *Distichlis spicata*, and tall, *Spartina alterniflora* from 2004 to 2011. Combined nutrient-enriched and reference creek data demonstrate significant increases in tall *S. alterniflora* and *D. spicata* with a coincident decrease in *S. patens* (*S. alterniflora* graph after Johnson et al. 2016).

Previously, Johnson et al. (2016) reported that there were no significant effects of long-term nutrient enrichment or year on the percent cover of tall *Spartina alterniflora*; however, in the present study by combining the data of the long-term enriched and reference creeks, we found a significant relationship of increasing cover of tall *S. alterniflora* (*F* = 17.876, *P* = 0.001) over time (2004–2011; Fig. 6A). In addition, significant relationships of increasing cover of *Distichlis spicata* (*F* = 6.096, *P* = 0.03) and decreasing cover of *Spartina patens* (*F* = 7.058, *P* = 0.02) were observed for the combined long-term enriched and reference marshes (Fig. 6B, C). Along with the sea level data for this coastal area, the species cover data suggest that the Plum Island experimental sites were receiving greater levels of tidal flooding and increased tidal creek volumes over the period of this study.

## Discussion

Marsh fracturing and subsequent slumping of vegetation into creeks in nutrient-enriched systems may in part be attributed to discontinuities in the vertical (w/depth) and horizontal (among vegetation zones) soil shear strength. The relatively lower soil shear strength in the root zone (10–30 cm depth) compared to sub-rooting depths in the interface vegetation of the long-term enriched systems may contribute to the fracturing and sliding of vegetated peat into the creek (Table 1; Fig. 1). In contrast, in the reference creeks the soil shear strength was more similar across the marsh landscape and there was no significant difference in soil strength between depths or vegetation zones. The soil strength of the rooting depth (10–30 cm) in the interface and creek bank zones was equal to or greater than the sub-rooting depths (40–60 cm; 70–90 cm) in the reference systems (Table 1). Production of roots and rhizomes was about 30 % greater in the reference creeks (Deegan et al. 2012: 579, ± 60 vs 387 ± 64 g/m^2^) and probably account for the positive or near-positive vertical differentials in the interface and creek bank zones in the reference creek systems (Fig. 2).

In a 13-yr nutrient enrichment experiment (surface broadcast of N-P-K fertilizer) of an oligohaline marsh located along the Tchefuncte River (Louisiana), a significant loss of root biomass, an increase in soil shear strength, and a sevenfold increase in shallow subsidence were reported (Graham and Mendelssohn 2014). The increase in shallow subsidence was attributed to reduced root biomass in the surface soils and the increase in soil strength to more resistant root structures and increased rooting depths (Graham and Mendelssohn 2014). In the Louisiana study, the shallow subsidence was apparently balanced by enhanced accretion rates (Graham and Mendelssohn 2014). In coastal areas where there are low sediment supplies such as the Plum Island Sound Estuary (suspended sediment concentration: about 3 mg/L), marshes exposed to accelerated sea level rise may have accretion deficits (Kirwan et al. 2010, Weston 2014).

Unlike many fertilization studies that surface broadcast or bury ammonium-based fertilizer into experimental plots, in this study nutrients were delivered as dissolved nitrate in the incoming tidal waters to the creek ecosystems, which provided for realistic nutrient loading levels (Johnson et al. 2016). We reported the highest soil strengths at sub-rooting depths (40–60 cm; 70–90 cm) and approximately 40% lower soil strengths at shallow depths (10–30 cm) in the interface zone of the long-term enriched marshes (Table 1), which represented a significant discontinuity in vertical shear strength. Longer exposure time of nutrients in the interface vegetation zone relative to the high marsh may have accelerated microbial decomposition, reduced root development, and contributed to fracturing and sloughing of peat into the creek.

The interface vegetation zone, primarily composed of *Spartina alterniflora, Spartina patens*, and *Distichlis spicata*, is the area on the landscape where the low marsh zone dominated by *S. alterniflora* transitions into the high marsh zone dominated by *S. patens.* The root zone of *S. patens* is described as an extensive, interwoven network of fine and coarse roots, maximizing biomass at shallow depths (about 4–8 cm; Connor and Chmura 2000), and the plant is able to develop aquatic roots that form into a fibrous network just above the existing marsh surface (Nyman et al. 2006). *Spartina patens* is sensitive to increased levels of nutrients and to the extent and frequency of inundation (Gleason and Zieman 1981, Emery et al. 2001, Nyman et al. 2006, Watson et al. 2016). Therefore, *S. patens* may be susceptible to increased inundation associated with rapid sea level rise in the nutrient-enriched interface zone. In contrast, *S. alterniflora*, the dominant plant in the low marsh, has extensive horizontal, large-diameter rhizomes and extensive, deep-dwelling (0–30 cm) active roots with well-developed aerenchyma tissue, along with metabolic adaptations, which together allow colonization at low elevation levels and provide tolerance to anoxic soil conditions (Gleason and Zieman 1981, Mendelssohn et al. 1981). However, reduced redox potentials and increased sulfide accumulations are associated with increased soil waterlogging, as might have occurred in the interface zone, which could reduce the growth of *S. alterniflora* (Mendelssohn and McKee 1988).

Increases in the soil % OM may be attributed to increases in biomass of benthic algae, bacteria, and other microbes in the long-term enriched creeks (Pascal and Fleeger 2013, Pascal et al. 2013), and/or to a loss of mineral material in the highly decomposed and eroding enriched marsh soils. The mean dry bulk density (0–35 cm) of the long-term enriched marsh soils (*n* = 20 cores; 0.44 ± 0.012 g/cm^3^) was significantly lower than that of the reference creeks (*n* = 20 cores; 0.48 ± 0.012 g/cm^3^; R. S. Warren, *unpublished data*, two-tailed *t*-test, *t* = 2.151, *P* = 0.033). The increase in the soil % C, N, and OM may contribute to the reported increases in microbial decomposition and denitrification rates (Koop-Jackobsen and Giblin 2010, Deegan et al. 2012) and soil respiration rates (this study) in the longterm enriched creek systems.

Generally, highly decomposed peat and decaying roots in the soil column will reduce soil strength (Swarzenski et al. 2008, Huat et al. 2009). The more sapric marsh soils in the longterm enriched marshes had higher water content and increased fine OM particulates, which may have reduced the frictional shear strength of the soils and increased fracturing with the rise and fall of the tides, resulting in shear failure of channel banks (Deegan et al. 2012).

The observed patterns of increasing tall *S. alterniflora* and *D. spicata* cover and decreasing cover of *S. patens* in both the long-term enriched and reference marshes (Fig. 6) support the hypothesis that accelerated sea level rise was increasing inundation of the Plum Island Sound marshes during our study. At least two factors suggest that increased inundation of sediment-poor water was an important driver of tidal creek bank failure. First, we found a trend toward reduced shear strength in the creek bank in comparison with the interface and high marsh zones, in both the enriched and reference systems. This may in part be attributed to higher water content of the frequently flooded creek bank soils, a factor that has likely increased over time with increased inundation associated with sea level rise. Second, studies of hydraulic geometry have suggested that increased inundation enlarges tidal channel prism volumes, which force shifts in channel dimensions to accommodate larger water volumes and may contribute to destabilization of the channel bank (Friedrichs 1995, D’Alpaos et al. 2010). Furthermore, in the enriched marshes the increase in inundation would increase the nutrient exposure time of the creek bank, stimulating decomposition processes, which could exacerbate the adverse effect of nutrient addition alone, possibly contributing to marsh fracturing and slumping.

The long-term enriched marsh displayed significantly higher creek bank fracturing and slumping compared to the reference marsh, in terms of fracture density (3 vs. 1 per 50 m), the percent of creek bank with fractures (30 vs. 7%), and fracture length (15 vs. 3 m; Deegan et al. 2012). It is an open question whether observed creek bank fracturing and slumping in the reference marshes were occurring at natural rates or at an accelerated rate due to rapid sea level rise and associated increases in inundation (Roman et al. 1997). At creek bank slumping locations in the long-term reference marsh, we measured a mean soil respiration rate of 2.43 ± 0.23 μmol·m^−2^·^−1^ (*n* = 5), a 27% increase in respiration rate compared with the mean long-term reference marsh rate (Fig. 3) and similar in magnitude to the short-term enriched marsh rate (2.94 ± 1.05 μmol·m^−2^·^−1^; n = 8). Increased soil respiration rates at slumping areas may also reflect an increase in marsh surface area exposed to air during the ebbing tide, fueling aerobic decomposition.

Results in this present study argue that discontinuities in vertical (with depth) and horizontal (across vegetation zones) soil strength likely contributed to the channel bank failures that have been observed in nutrient-enriched systems. Tide gauge data revealed a pattern of rapid sea level rise for the period of the study, and changes in plant distribution patterns suggested increased inundation over time at both the enriched and reference sites. Longer exposure times to nutrient-enriched waters and increased hydraulic energy associated with sea level rise may have exacerbated creek bank fracturing and slumping. The combined effects of coastal eutrophication and accelerated sea level rise may help drive marsh losses in many coastal areas worldwide where nitrogen loads and sea levels are both rising. The results of this study strongly support that additional research is needed to better understand the interactions of nutrient enrichment and sea level rise on marsh soil strength and stability.

## Figures and Tables

**Table 1.** Soil shear strength (kPa) measurements (units ± SE) for reference and enrichment at 10 cm increments from 10 to 90 cm depths, and means (reported in boldface) of the 10–30, 40–60, and 70–90 cm depth intervals across creek bank, interface, and high marsh vegetation zones.

Parameters	Depth (cm)								
	10	20	30	40	50	60	70	80	90
Short-term reference									
Creek bank	19.3 ± 2.6	25.8± 5.4	25.5 ± 2.5	24.5 ± 2.9	21.7± 2.7	20.2 ± 3.6	22.0 ± 4.6	27.5± 4.0	34.8 ± 5.7
		**23.5 ± 3.5**			**22.2 ± 3.1**			**28.1 ± 4.8**	
Interface	37.8 ±7.6	23.5 ± 1.8	23.3 ± 1.5	23.3 ± 1.7	26.3 ± 2.8	27.5 ± 1.6	27.5 ± 2.1	29.0 ± 2.8	31.3 ± 4.2
		**28.2 ± 3.6**			**25.7 ± 2.0**			**29.3 ± 3.0**	
High marsh	28.0 ± 5.0	24.8 ± 4.6	24.0 ± 3.6	21.5 ± 3.7	21.0 ± 2.3	22.0 ± 3.3	25.3 ± 2.0	22.8 ± 2.9	23.8 ± 3.7
		**25.6 ± 4.4**			**21.5 ± 3.0**			**23.9 ± 2.9**	
Long-term reference									
Creek bank	22.3 ± 4.6	26.5 ± 6.2	23.8 ± 3.8	17.5 ± 2.3	20.0 ± 3.4	18.0 ± 3.7	19.3 ± 3.2	22.8 ± 5.3	24.3 ± 4.9
		**24.2 ± 4.9**			**18.5 ± 3.1**			**22.1 ± 4.5**	
Interface	25.0 ± 3.0	21.3 ± 2.7	21.8 ± 2.3	21.0 ± 2.1	25.5 ± 2.6	24.0 ± 2.6	23.8 ± 2.0	28.0 ± 2.4	30.0 ± 3.3
		**24.8 ± 2.6**			**24.1 ± 2.1**			**27.3 ± 3.2**	
High marsh	25.8 ± 2.1	25.8 ± 3.6	23.0 ± 2.0	23.1 ± 2.4	23.4 ± 1.8	25.8 ± 2.1	28.0 ± 3.0	27.0 ± 3.0	27.0 ± 3.6
		**22.7 ± 2.7**			**23.5 ± 2.5**			**27.3 ± 2.6**	
Short-term enrichment									
Creek bank	19.0 ± 2.2	24.5 ± 2.7	26.3 ± 3.5	22.5 ± 2.9	22.1 ± 2.5	20.8 ± 4.3	25.4 ± 4.7	29.3 ± 4.1	29.5 ± 5.4
		**23.3 ± 2.8**			**21.8 ± 3.2**			**28.0 ± 4.7**	
Interface	18.0 ± 1.7	18.6 ± 1.9	19.4 ± 3.3	20.3 ± 3.5	23.8 ± 4.3	30.5 ± 1.9	27.8 ± 3.6	24.8 ± 3.8	27.5 ± 3.8
		**18.7 ± 2.3**			**24.8 ± 3.2**			**26.7 ± 3.8**	
High marsh	31.3 ± 7.8	25.1 ± 3.1	27.3 ± 2.5	30.3 ± 1.4	27.4 ± 4.6	21.5 ± 2.8	22.8 ± 4.6	18.6 ± 3.1	22.5 ± 2.9
		**27.9 ± 4.5**			**26.4 ± 1.9**			**21.2 ± 3.5**	
Long-term enrichment									
Creek bank	18.8 ± 4.0	27.3 ± 4.0	24.0 ± 3.5	22.5 ± 3.3	24.2 ± 3.1	21.0 ± 4.2	21.5 ± 3.5	21.5 ± 3.5	25.5 ± 2.7
		**23.3 ± 3.8**			**22.6 ± 3.6**			**22.8 ± 3.3**	
Interface	19.0 ± 2.0	17.8 ± 2.0	19.8 ± 1.5	27.3 ± 1.2	30.5 ± 2.7	34.0 ± 2.1	31.8 ± 2.5	31.2 ± 2.9	34.0 ± 2.7
		**18.8 ± 1.8**			**30.6 ± 2.0**			**32.3 ± 2.7**	
High marsh	19.9 ± 1.8	22.5 ± 1.9	32.0 ± 3.1	29.3 ± 1.9	30.5 ± 2.1	31.8 ± 2.2	33.3 ± 3.6	29.0 ± 3.1	30.8 ± 3.1
		**24.8 ± 2.2**			**30.5 ± 2.1**			**31.0 ± 3.3**	

*Notes:* Abbreviations are creek bank, creek bank tall *Spartina alterniflora*; high marsh, *Spartina patens*-dominated high marsh; interface, mixed *S. alterniflora, S. patens*, and *Distichlis spicata* vegetation between creek bank and high marsh. Mean shear strengths for zones were calculated across sampling locations (*n* = 8 per treatment). Mean 30 cm depth interval shear strengths were calculated by averaging 10 cm depth measurements (10–30, 40–60, 70–90 cm) for each.
